# Dr. Breed’s Reply to Dr. McElroy

**Published:** 1869-12

**Authors:** 


ARTICLE XLIII.

### DR. BREED'S REPLY TO DR. McELROY.

Since my report on Ileitis, in the October number of the *Chicago Medical Examiner*, I have received several letters from medical men living in different parts of the country, in regard to the case.

While all seem to agree in commending the report in a general way, each has some suggestion to make in reference to some minor point, where he thinks the paper might have been improved. In comparing these letters I find that what one criticises, another praises, and *vice versa*—no two agreeing in condemning the same thing; so that I find it impossible to please all, and, indeed, have not quite satisfied myself. Dr. Holton, an intelligent physician, of Buda, Ill., thinks I should have given more prominence to the peritoneal inflammation, evidenced by the extensive adhesions, as, to his mind, that was the principal lesion to be considered.

Dr. W. H. Nance, a successful and experienced physician of Vermont, Fulton County, Ill., writes me as follows:

"I will say, Doctor, that I am much pleased with the "*report*," and the conclusions drawn from it, and presume that they are correct, in the main, and that you are right in your conclusions respecting the results emanating from inflammation of the muscular coat of the small intestines. I am not sure, however, that in your concluding paragraphs, where you are summing up the fatal results of such a condition, that you have made the happiest selection of a word to represent that condition, where you speak of a portion of the alimentary canal being *paralyzed.*

"That all normal function is suspended, is admitted; but is not that suspension the result of a condition the very reverse of paralysis? Paralysis is a loosening, relaxing, or letting go, from want of nervous force, or energy. But this intestinal condition is the result of a hyper exaltation of the nervous and muscular powers, of the part, blown into the intense heat of inflammation, represented as being *seized* and held as with the hand of some etherial Titan."

Dr. Z. C. McElroy, a medical philosopher of Zanesville, O., in a carefully written "Memoir," on "The forces of organic life," reviews this report from a different stand-point, and, in the light of certain philosophical, physiological, and hypothetical principles laid down by him, reaches a conclusion that it was *simply* a case of lead-poisoning.

Now, as all these suggestions are made in a courteous and friendly way, and intended to enure to my benefit, and promote the science of medicine, I am exceedingly thankful for them, and will try to profit by them. That this report should have thus called out this discussion, upon the interesting questions involved, is neither surprising nor displeasing to me. Indeed, I have no doubt that, in the end, I shall not receive the *least* of the *benefits* from it.

In offering a few additional remarks upon the same subject, it may as well be admitted, that, while working up the report from the symptoms, history, and autopsy, my mind was, for a time, undecided as to how best to interpret the phenomena. The leading symptoms, during the illness, were those of obstruction. The autopsy, however, revealed the effects of extensive and severe inflammation, involving all the coats of the small intestines, together with such signs as led to the belief that the cerebro-spinal and ganglionic masses were seriously implicated in the disease. While my mind was thus undecided, halting, as it were, between two opinions, *viz.:* Whether I should go back of the inflammation, which was an undoubted element in the disease, and interrogate the nerve centres for the prime cause of the difficulty, or, on the contrary, confine my speculations to the former, and hold inflammation responsible for the trouble; the question of *lead-poisoning* came up for consideration.

The occupation, habits, and several striking peculiarities of the case, conspired to direct my attention to this particular *point.* I was told that the patient was much in the habit of holding *type in his mouth* while at work. This was a leader. The symptoms that led me to suspect the integrity of the nerve centres were, a *decided* and *unmistakable* absence of arterial tensions, and the partial paralysis of the abdominal muscles, so as not to exhibit the usual reflex movements in emesis and defecation; and the *post mortem* displayed, an *atonic* condition of the muscular fiber of the intestinal walls, indicated by a distended condition of the empty intestines, where we usually find them collapsed.

I first directed my attention to this question. In lead-poisoning, are the intestines, at the seat of the disease, distended, dilated, or are they, on the contrary, collapsed, or contracted? After a careful investigation of this subject, I came to the conclusion that there was a vast preponderance of testimony in favor of the opinion that, where there was any pathological appearance at all, a *contracted* and *collapsed* condition were those *generally found* at the seat of the disease. The arteries were also, in lead disease, smaller than natural, from the same general contractile condition, affecting the arterial coats, and, moreover, the tissues affected were found generally *pale*, *exsanguined*, and more or less *wasted.* The disease, "tabes saturnina," is supposed to be due to this constringing influence of the bloodvessels.

While DeHaen, Merat, Eberly, Wood, and Letherby, concur in the belief that the *contracted* or *collapsed* state of the empty bowel, at the seat of the disease, is the pathological condition to be looked for, Sir G. Baker, Andral, Townsen, West, and Louis have *often* found *no alterations whatever* in the *intestines.* In a case which terminated with the symptoms of saturnine encephalopathia, *viz.:* delirium insensibility, and tetanic convulsions, Empis and Robinet found no anatomical alterations of any importance. Lead was discovered by incineration in the brain and liver.

In Letherby's case, lead was freely discovered in the stomach, in the brain, muscles, liver, intestines, blood, and in the serum of the cerebral ventricles. The stomach and intestines were *pale*, and the latter were contracted, and in some places invaginated. Pereira says, in regard to the constitutional effects of small doses of lead, that the arteries become reduced in size and activity, and that the *pulse becomes* slower and *smaller*, from which I infer that the preparations of lead give rise to a contracted state of the bloodvessels. How did these symptoms correspond with my case? Why, the muscular coat of the small intestines, besides showing such signs of inflammation as thickening, a deep red, or purple color, the muscular fibers were evidently in an atonic, inactive, or paralyzed condition, so that they were quite unable to propel the contents of the tube onward. They had lost their contractile power. This was a prominent feature in the case, and one that I believed was the key to the explanation of the unusual phenomena in the symptoms, and I desired, therefore, particularly to emphasize it. This want of contractile power in these mucular fibers was the cause of the symptoms of obstruction. There was no other apparent cause to be found. Was this suspended function due to some lesion in the nerve centres, or was it a result of the inflammation of the muscular coat itself? This was the question. I acknowledged that I was not quite satisfied on this point; but, in either case, the symptoms of obstruction would be the same, and, as the former hypothesis involved a somewhat speculative question, and the latter would fully account for all the symptoms, and, moreover, not finding the symptoms to correspond with those of lead-poisoning, I concluded to adopt the simpler explanation, and not go behind the inflammation in the structures for the proximate cause.

While my mind was perplexed on this subject, I was assisted, perhaps, by Prof. Gross' definition of disease, quoted by Dr. McElroy, in reply to Dr. Hendrick, in the July number of the *Chicago Medical Examiner*, for 1868. Dr. McElroy says:—

"First. What is disease? Disease is not, as it was formerly supposed to be, a special entity, a particular essence, a some thing vague, intangible, mysterious, but simply *a departure* from the normal standard, a change in, or of a part, brought about by the *perverted* action of its *circulation*, *innervation*, and nutrition; and modified by function and structure. Nearly every disease, whatever its nature or site, is *essentially* an *inflammation.* Even in what are called the *neuroses*, or nervous affections, *inflammation* generally plays a conspicuous part."* By carefully scanning this definition, it will be seen that disease is simply an *abnormal condition*, an obvious change, in a part, brought about by a *perverted* action of the *circulation*, *innervation*, or *nutrition.* Dr. McElroy seems to endorse this definition, and hence, he will be likely to excuse me for not being ready to call the disease by some name that would aim to ignore the *inflammation*, and fix the trouble upon the lesion of *innervation.* Let it be remarked that the *perverted* action *here* is not the disease, but the proximate *cause* of the *disease.* Now,

* Prof. Gross, "Now and Then," published address. 1867.

suppose Dr. McElroy does succeed in establishing a fact in this case that the trouble could be traced remotely to the gray matter of the "nervous masses," it can only be called a *perverted action* (the cause), resulting in *inflammation* (the result, or disease,) according to his own definition. If all diseases are, moreover, essentially an *inflammation*, I could not have been very far wrong when I denominated the disease an inflammation of a particular structure. But I do not desire to split hairs with the Doctor, for I am too well pleased with his paper for that.

That the reader may have a fuller view of the matter, and enable him the better to understand my reasons for not ascribing the disease to lead, I here submit the principal symptoms of the two diseases, in parallel columns. The symptoms of lead-poisoning in the first, and those reported in my case of ileitis, in the second:—

POST MORTEM APPEARANCES IN BOTH.

| *Symptoms of Lead-Poisoning.* | *Symptoms of Ileitis.* |
|---|---|
| Intestines generally contracted. | Intestines dilated. |
| Inflammation not characteristic. | Inflammation severe. |
| Muscular fiber *pale*, exsanguined, and wasted. | Muscular fiber red, purple, atonic, and thickened. |
| Skin *generally* hot and dry. | Skin *cool* and *moist*. |
| Much *pain* and colic. | Very little pain. |
| Signs of spasms. | No spasms. |
| Convulsions. | No convulsions. |
| Gums, pale bluish line. | |
| Tongue flabby, and tremulous. | Tongue covered with dark, slimy coating. |
| Mouth dry. | Mouth moist. |
| Urine scanty, and high colored. | Urine free, not particularly high colored. |
| Face pale, dingy, and sallow. | Face flushed and livid. |
| Motor power impaired. | Sensory power more injured. |
| Weakness of wrists and joints. | Normal. |
| Extremities cramped. | Normal. |
| Pressure gives relief to pain. | Pressure not well borne. |
| Muscles of chest spasmodically contracted. | Muscles of abdomen *paralyzed*. |
| Pulse slow, and artery small. | Pulse slow, and soft, undulating, and artery large. |

| | |
|---|---|
| Loss of sensorial function. | Normal. |
| Palsy of extremities. | Normal. |
| Vomiting bilious matters. | Vomiting bilious and stercoraceous matters. |
| Hardened feces in passages. | Feces thin and aqueous. |

Such are the most prominent symptoms, and anatomical changes found in lead disease, as contrasted with those reported in the case denominated ileitis.

After as full and careful an examination of the symptoms, morbid anatomy, and general history of lead-poisoning, as I was able to make, and comparing them with the case in hand, *pro* and *con*, I determined on the name "Ileitis," as the best term I could think of, to indicate the nature and pathology of the affection, I concluded, therefore, to make no mention of lead, as cutting any figure in the disease; but I aimed, nevertheless, to employ such phraseology as would convey to the mind all the important features of the case. I expected every physician who read the report *carefully* would form his own opinion of the diagnosis, and, therefore, I concluded to send it forth to meet its fate, while I anxiously waited the *denouement*, whatever it might be.

Whether I was right or not, I do not *now* know. Those who, with myself, looked upon inflammation of the coats of the bowels as the *essential* and most *important element* in the *disease*, will now see, and understand why I used the term "*paralysis*," as I did; while those who, from the symptoms and autopsy, were led to infer some serious lesion of the nerve centres, will, at least, allow the fidelity of the description. It may be remarked in this connection, however, that while studying Dr. Abercrombie's observations on ileus—(see diseases of the abdominal viscera)—I found that he declared, in opposition to all the above authors, that the *collapsed portion* of the *empty intestine*, is the *natural condition*, while the distended portion is the primary seat of the disease; the distention arising from a paralytic condition of the muscular fiber, whereby it is unable to contract and propel the contents of the tube onwards through it. Now this view seems very probably the correct one, if we apply the same hypothesis to lead colic, since the action of lead upon the muscular fibers of the intestines is doubtless of the same kind as that exerted upon the fibers of the voluntary muscles. This hypothesis, moreover, is greatly strengthened when we consider the therapeutic action of *alum* in *curing lead colic.*

In most, if not all, of our works on therapeutics, there is a striking analogy between the recorded symptoms of lead, and alum, when administered internally in small doses. Nevertheless, alum is represented as the best remedy in lead colic. This looks unphilosophical, and should be looked into. We do not cure diseases on the Homœopathic theory of "*similia similibus curantur*," but on that of "*contraria contrariis curanter*," instead. Now, Dr. Copeland has actually ascribed the curative effect of alum in lead colic, to its power of exciting the contraction of the partially paralyzed muscular coat of the small intestines in this disease. If he is correct in thus regarding these two remedies as antagonistic, and antidotal, in their effects on the system—that, while one has a tendency to relax, and paralyze the intestinal, arterial, and voluntary muscular fiber, the other has a directly opposite tendency; then will the paradox be explained, and the philosophy of our medication be vindicated. I feel under deep obligation to Dr. McElroy, and the other medical gentlemen, for raising these questions in the manner they have, and thus stimulating inquiry on this subject. With my eyes half open to these facts, I felt sure that there was a condition in the case justifying the term paralysis, yet I felt unwilling to launch out into unknown seas, and indulge in fruitless speculations upon the mysteries of innervation. That there was some fault, some abnornal condition in these nerve centres, interfering with their acknowledged function in keeping up that tireless state of muscular tension throughout the body, I felt quite confident. The atonic condition of the muscular fiber in the alimentary canal, in the coats of the arteries—in the abdominal and respiratory muscles, all clearly indicated this want of nervous power. This striking peculiarity first awakened my suspicion that there was some toxic agent circulating in the blood, and thus weakening the secret springs of life. Had the symptoms of lead-poisoning corresponded with these notable facts, I should have attributed the difficulty to that cause.*

I am, by no means, unobservant of the fact that these nerve centres are, especially in this climate, frequently at fault, and seem to constitute one of the first, and most important links in the chain of morbid actions in very many diseases. In a report, as Chairman of the Committee on Practical Medicine, read before the "Military Tract Medical Association," and published in the April number of the *Chicago Medical Examiner*, for 1868, I devoted considerable attention to the importance of the cerebro-spinal and ganglionic systems of nerves in the diseases of Northern Illinois.

In this report will be found enunciated views, which seem peculiarly applicable in this connection. The importance of the subject must be my excuse for repeating them here.

When the nerve centres presiding over nutrition, digestion, and the intestinal secretions generally, from *any cause* (and they are subject to be sympathetically modified in their molecular actions by the slightest and most varied impressions upon any portion of the body) become too much depressed, irritated, or *in any way* so modified in the delicacy of their normal structures as to unfit them for furnishing the stated necessary nervous supply, *disordered digestion is the result*, either diarrhœa or *constipation*." Again, I quote from the same report: "When the nature and *modus operandi* of these delicate structures are better understood in maintaining the *solidarity* of the whole human economy, the part they play in the delicate processes of innervation, sensation, digestion, secretion, elaboration, nutrition, calorification, growth, extension, and repair, respiration, disintegration, excretion, locomotion, and ratiocination, we may begin to conceive something of the importance and magnitude of this intricate subject."

Again: "When it is borne in mind that, in this climate, especially, the *onus* of disease is often thrown upon these struc-

* That inflammation in a muscle is quite competent to arrest its power of contraction, many familiar examples might be given, aphonia in laryngitis is one good illustration.

tures, and that it is in these nerve centres that the *nerve force* is correlated into *cell* action—*cellulation:* (the primal and fundamental process of all organized structures,) secretion, etc., that it is here, indeed, that the nerve force *seems* to be generated, and that the nerve centres *altogether* preside over every physical phenomena in the body, it will not seem so strange that even *slight molecular changes* in these delicate tissues should crop out in grave and formidable symptoms of disease; and, more especially still, when it is remembered that these nerve centres *first* feel the noxious or depressing effects of any toxic agent in the circulation, that they, more than any other organ of the body, therefore demand for their normal action pure, oxygenated blood, and healthy food, free from any deleterious agents, we shall be able to understand why they are so frequently at fault." Hence it will be seen, I hope, that there is no inconsistency in the hypothesis of *a perverted* action of these nerve centres, even though we may not be able always to designate the precise cause of the perversion.

Should it be decided, however, after all, that Dr. McElroy is correct in his opinion of the principal agency of lead in the case reported, then will it appear still more probable, in the light of all the phenomena in this case, that Dr. Abercrombie is right in his view of the pathology, and Dr. Copeland, in the *modus medendi* of alum in the cure of lead colic; and that all the other eminent pathologists cited are mistaken.

However this question may be viewed, it is an interesting subject of inquiry; and with the ultimate decision I shall be content.

